# The interventional therapy for diabetic peripheral artery disease

**DOI:** 10.1186/1471-2482-13-32

**Published:** 2013-08-20

**Authors:** Nian-feng Sun, Ai-ling Tian, Yu-ling Tian, San-yuan Hu, Li Xu

**Affiliations:** 1 Department of General Surgery, Qilu Hospital of Shandong University, Jinan 250012, China; 2 Department of the Surgery, Affiliated Jinan Central Hospital of Shandong University, Jinan 250013, China

**Keywords:** Diabetic, Peripheral artery disease, Angioplasty, Interventional operation

## Abstract

**Background:**

Diabetic peripheral arterial disease is the main cause of lower limb amputation in patients with diabetes. To summarize the technique and experiences and evaluate the clinical effects of blood vessel intervention operation on diabetic peripheral artery disease.

**Methods:**

81 patients with diabetic peripheral artery disease from October 2007 to September 2011, 81 cases of the observation group were treated by balloon PTA. By adopting the Seldinger puncture technology, intubation was placed into a cobra catheter or a pig tail artery catheter and directed to the ipsilateral lower extremity artery. A guidewire was used to reach the lesion part of patients and a long balloon with a diameter of 4–6 mm was used to expand the artery with a pressure of 6–10 atm.

**Results:**

81 patients in the observation group received the PTA surgery. The technical succesful rate was 100%, no complication happened. The skin temperature increased after treatment. The blood supply improved significantly. The pulsation of the foot dorsal artery was strengthened. The numbness and pain symptoms were moderated significantly. We observed better results in the observation group in lower limb vessel diameter and foot ulceration healing. None of the patients received amputation surgery. Its short-term effects were satisfactory.

**Conclusion:**

PTA is a feasible technique for diabetic peripheral artery disease. It has great clinical significance in treating diabetic peripheral arterial disease. Although its short-term effects is satisfactory, the long-term effects is necessary for follow up.

## Background

Peripheral arterial disease (PAD) is a group of disorders characterized by narrowing or occlusion of the arteries resulting in gradual reduction of blood supply to the limbs. PAD may be asymptomatic until it reaches an advanced stage [1] Diabetic peripheral arterial disease is the main cause of lower limb amputation in patients with diabetes. Patients with diabetic peripathic arterial disease are at high risk of increased morbidity and mortality from cardiovascular diseases. Factors, such as age and the duration of the disease, will increase its incidence and risk of death, and uncontrolled infection often leads to amputation [2,3] worldwide and that patients with diabetes are at excess risk of developing PAD, the implications of the problem are enormous [4].

The Hoorn study found that the prevalence of ABI < 0.9 in individuals with normal glucose tolerance was 7% and increased to 20.9% in patients with diabetes, while a pilot study found a prevalence of asymptomatic PAD of 33% [5,6].

In the Rochester study, the cumulative incidence of PAD was 15% 10 years after the diagnosis of DM, which increased to 45% 20 years later [7]. Patients with diabetes have increased risk of lower-extremity amputations in comparison with non-diabetic subjects and the severity of PAD in DM assessed angiographically has been associated with major amputations [8,9]. The 5-year mortality in patients with diabetes and critical limb ischaemia is 30% [10].

The aims in the management of the patients with diabetic peripathic arterial disease are to improve symptoms and to prevent cardiovascular morbidity and mortality. Since traditional medicine conservative treatment has poor effects and vascular bypass surgery often causes large trauma, new treatments such as percutaneous transluminal angioplasty (PTA) and luminal stenting surgery have emerged in recent years. Both of these are endovascular treatments. Classically, the indications for a PTA in diabetic peripheral arterial disease are disabling claudication affecting quality of life after medical therapy has failed to improve symptoms, and critical limb ischaemia symptoms. With smaller surgical trauma and certain curative effect, these procedures have become the hotspots in the field of treatment of diabetic peripheral arterial disease.

From 2007 to 2011, endovascular treatments were used in 119 lower extremities of 81 patients with diabetes. The results showed that the treatment had good therapeutic effects.

## Methods

### Patients

From October 2007 to September 2011, 81 hospitalized patients with diabetic lower extremity arterial disease were chosen for the study. The patients had a indication, for example intermittent limping, rest pain and ischemic ulcer. Among them were 48 males and 33 females with ages ranging from 46 to 82 years old. The duration of diabetes varied from 6 to 26 years and the duration of diabetic peripheral arterial disease varied from 2 to 21 years. Patients were diagnosed with diabetic peripheral arterial disease as their lower extremity arterial ultrasound examination showed different degrees of segmental stenosis and block as well as foot lesions. A total of 43 patients were complicated with 59 diabetic foot ulcers, and they were segregated according to the Wagner classification [11]: 14 at level 1, 24 at level 2, 17 at level 3, and 4 at level 4.

There were 13 patients who also had cerebrovascular disease, 17 patients who also had coronary heart disease, 21 patients who also had hypertension, 15 patients who also had coronary heart disease and hypertension simultaneously, and 15 patients who also had peripheral neuropathy. All of their digital subtraction angiography (DSA) showed segmental stenosis (>50%) or short segmental lesions. All patients in the observation group received percutaneous transluminal angioplasty or luminal stenting surgery, and all were treated successfully. All patients were screened for the absence of anemia, chronic cor pulmonale, and other diseases which can cause hemodynamic changes.

### Statements

We confirmed that our study was approved by ethics committee of Qilu Hospital of Shandong University. We confirmed that informed consent was obtained from all study participants for our inclusion in our study.

### Therapeutic method

The blood sugar of patients were controlled in a stable range after some diet and drug therapy (Blood-fasting sugar 3.9 mmoL/L-6.1 mmoL/L). The groups were treated with improving microcirculation drugs (e.g., Ligustrazine Hydrochloride for injection, Xingding solution and Sodium Aescinate for injection), neurotrophic drugs (e.g., Mecobalamin tablets), and local dressing.

The surgical operation was performed using a large x-ray machine (Siemens, Germany). Eleven patients selected the antegrade puncture, 67 patients opted for the contralateral femoral artery puncture, and 3 patients chose the humerus artery puncture. By adopting the Seldinger puncture technology, intubation was placed into a cobra catheter or a pig tail artery catheter and directed to the ipsilateral lower extremity artery. Then, a step-by-step DSA was used to take angiography for each segmental artery separately.

**Figure 1:**
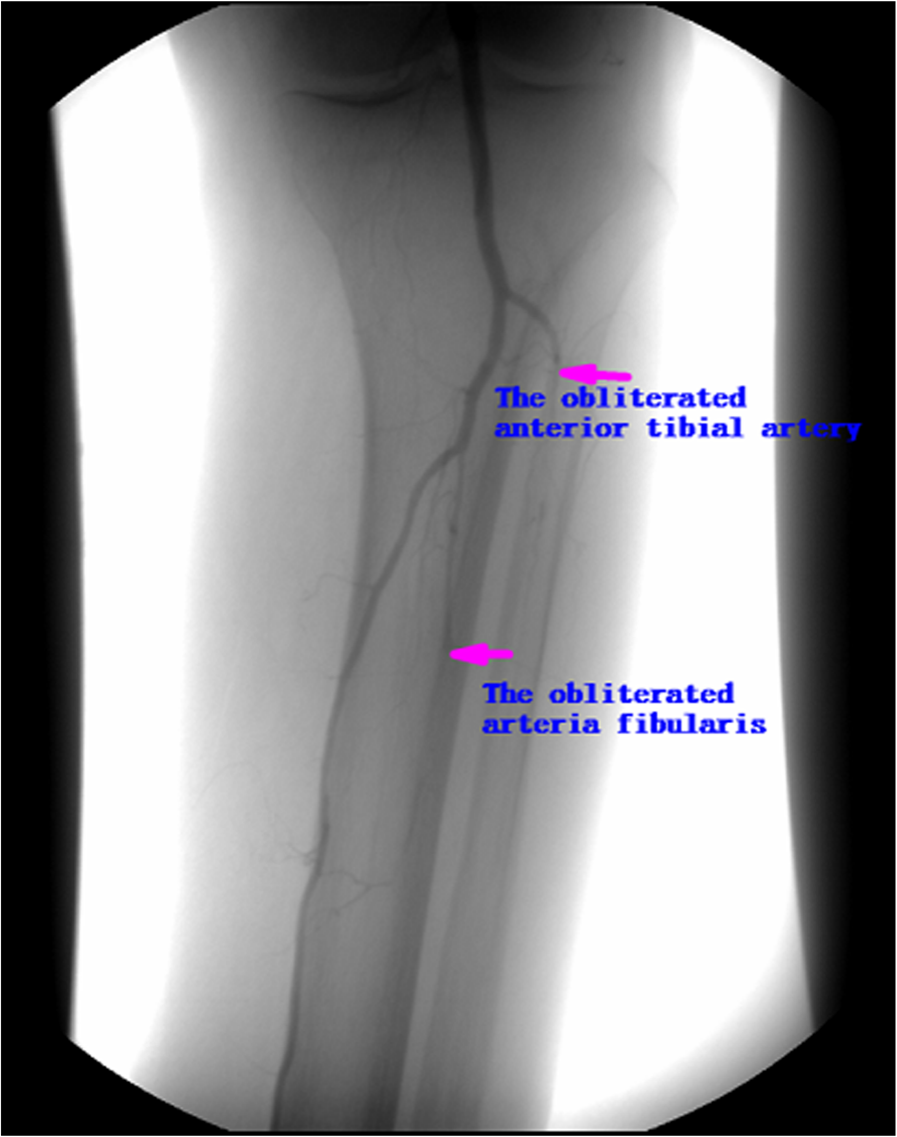
The arteriography picture prior treatment (The obliteration of anterior tibial artery and arteria fibularis).

**Figure 2:**
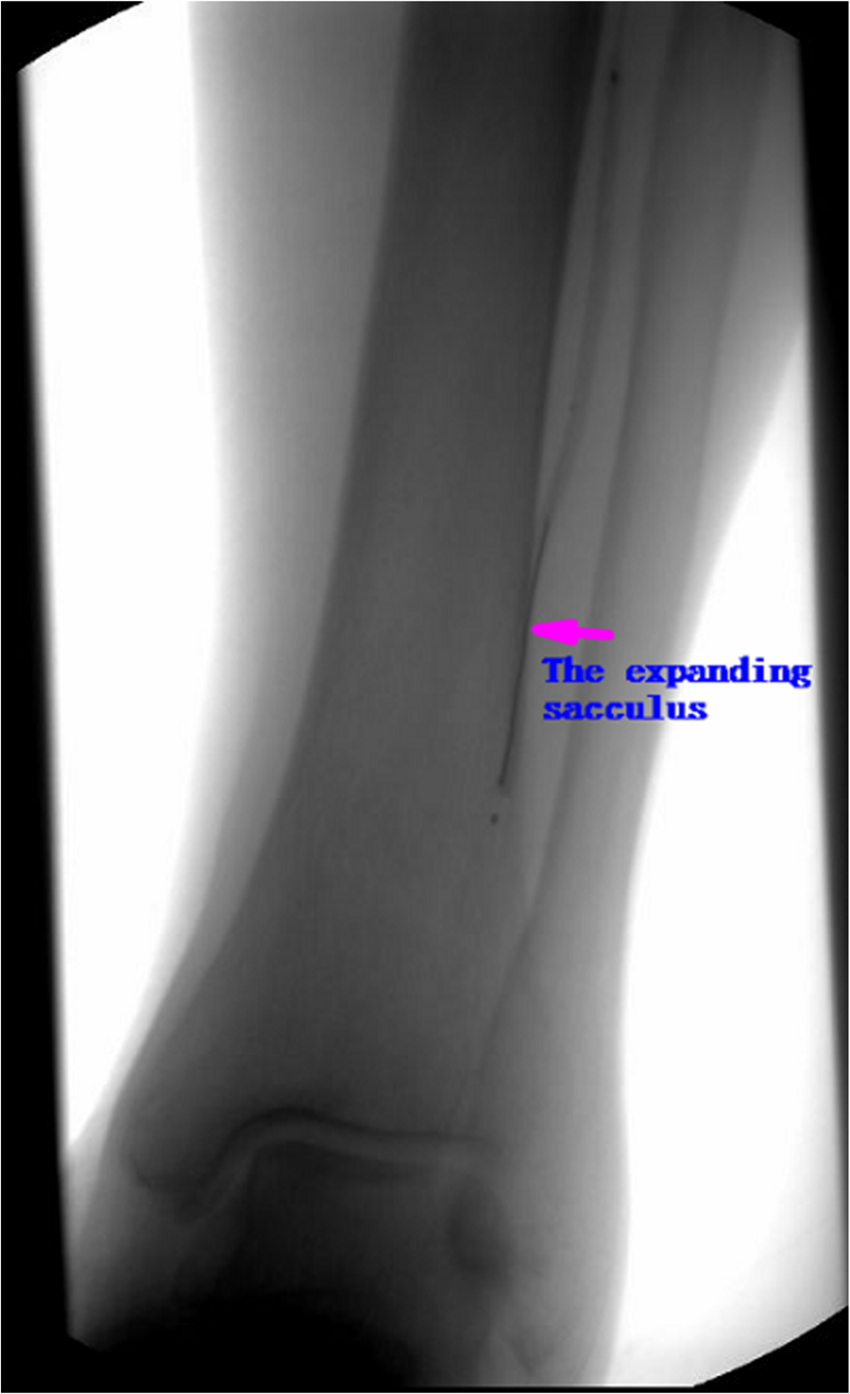
The arteriography picture in the operating (The sacculus was expanding the anterior tibial artery).

**Figure 3:**
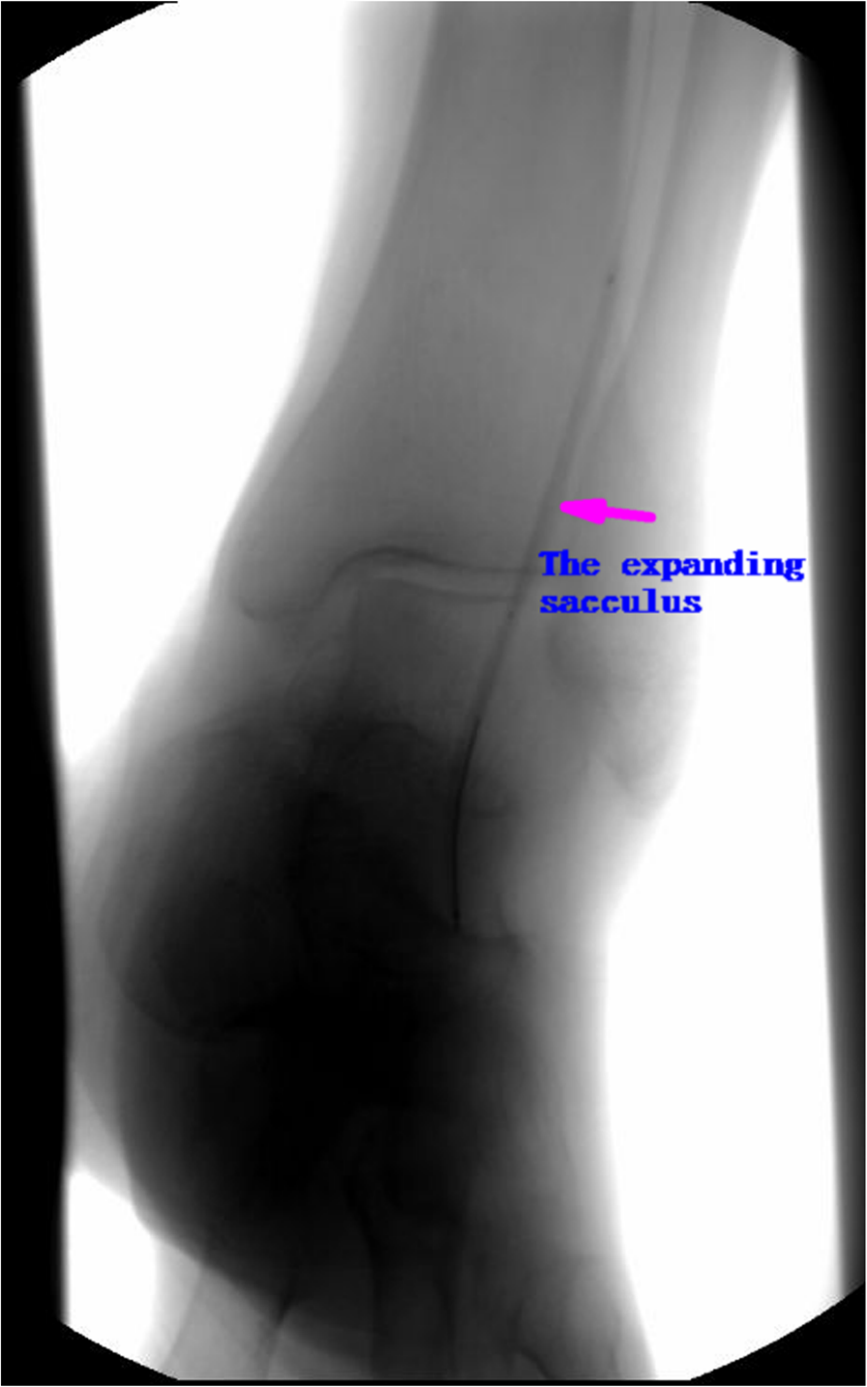
The arteriography picture in the operating (The sacculus was expanding the arteria fibularis).

**Figure 4:**
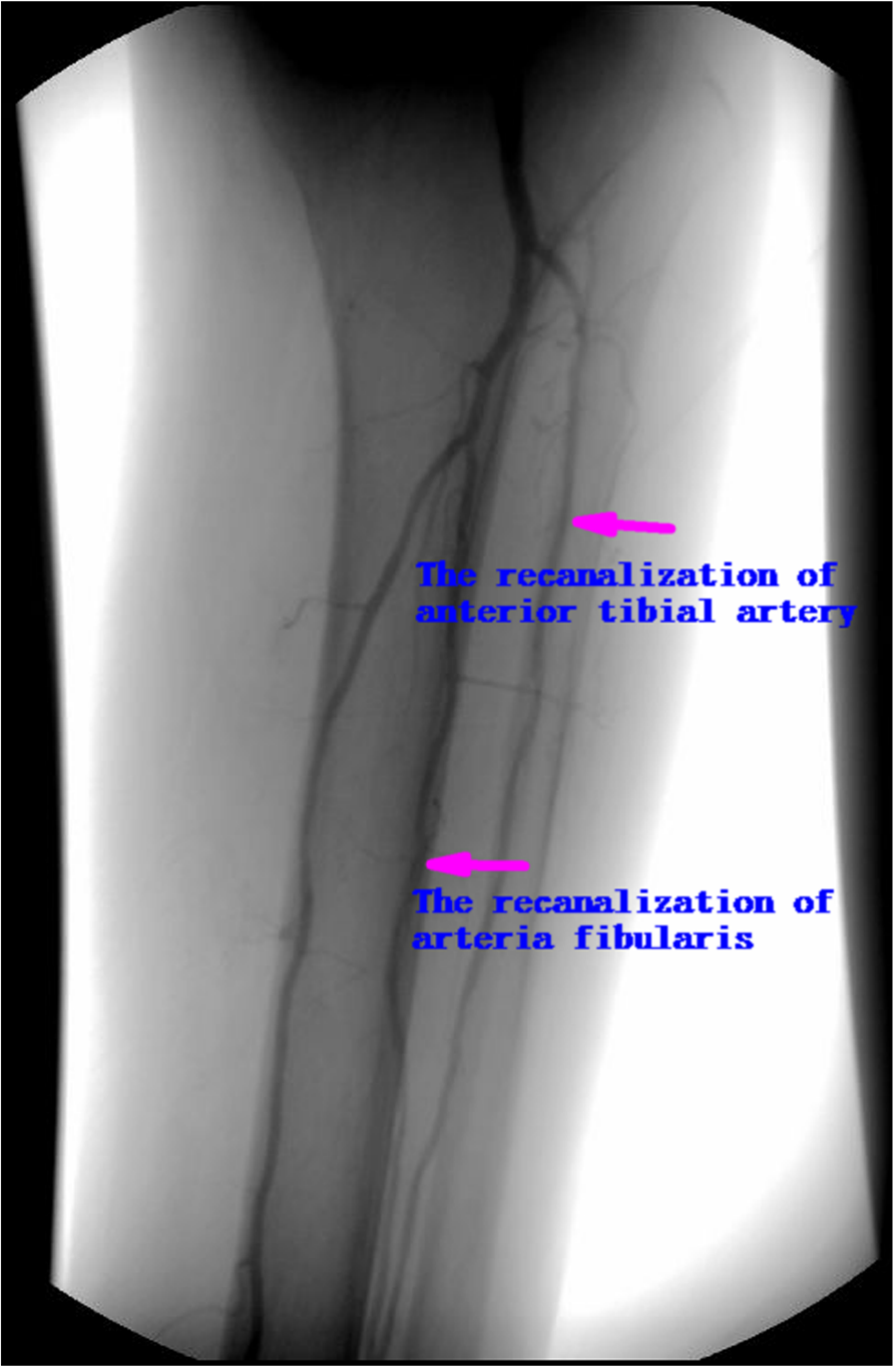
The arteriography picture post treatment (The recanalization of anterior tibial artery and arteria fibularis).

For patients with segmental stenosis or short segmental lesions in the superficial femoral artery or femoral profound artery, a 0.035 inch stiff type guidewire was used to reach the lesion part. A long balloon with a diameter of 4–6 mm was used to expand the artery with a pressure of 6–10 atm. If the stenosis is more serious or if the artery retracted after expansion, it was placed in a self-expandable stent of an appropriate model (Cordis, USA). For patients with infrapopliteal artery stenosis, a 0.014 inch stiff type guidewire was used. This was designed for tibial and peroneal artery disease. A deep long balloon with a major axis of 120 mm was used to get through all infrapopliteal arteries, including the most distant plantar arteries (Figures 1, 2, 3 and 4).

To prevent postoperative arterial thrombosis, a regular anticoagulant therapy was adopted: Clopidogrel Hydrogensulfate Tablets 50 mg, every day × 1, per os for six months; Bayaspirin 100 mg, every day × 1, for long-term use. In addition, symptomatic supportive treatment, such as blood sugar control, infection prevention, improving microcirculation, and local dressing, should be provided after surgery. Six months later, another vascular color Doppler ultrasound examination was performed.

### Evaluation indexes

Clinical symptoms, vascular ultrasonic changes, and foot ulcer healing conditions were observed and evaluated. The following observations have been concluded: a) Clinical symptoms changes included skin color, pain, temperature sensation, and pulsation of foot dorsal artery; b) The lower extremity vascular color Doppler ultrasound examination showed the changes in arterial diameter; c) The size, secretion, and wound granulomas condition were observed separately at 10, 30, and 60 days after the surgery.

### Statistical methods

For this study, SPSS 11.0 statistics software analysis technology was adopted. The research was made with a two-factor factorial design. The result can be analyzed with variance analysis.

## Results

In the observation group, 81 patients received the PTA surgery. Out of the 81 patients, 35 added a self-expandable stent (Cordis Company) after balloon expanded. This resulted to a success rate of 100%.

For patients in the observation group, the skin temperature increased after treatment. The blood supply improved significantly. The pulsation of the foot dorsal artery was strengthened. The numbness and pain symptoms were moderated significantly. In all, its short-term effects were satisfactory.

The vascular ultrasound results of the observation group showed that the vascular diameters have increased significantly compared with the data taken before treatment as well as compared with the control group. It had an efficiency rate of 100% (Table 1).

**Table 1:** Comparison of diameter changes at the narrowest point of the lower extremity artery under vascular color Doppler ultrasound examination before and after treatment

**Time of therapy**	**Arteria cruralis(mm)**		**Popliteal artery(mm)**		**Arteria dorsalis pedis(mm)**	
	**L**	**R**	**L**	**R**	**L**	**R**
prior treatment	7.11 ± 0.14	7.12 ± 0.15	5.14 ± 0.36	5.11 ± 0.26	1.22 ± 0.11	1.21±0.32
post treatment	7.68 ± 0.11	7.66 ± 0.41	5.70 ± 0.45	5.67 ± 0.42	1.75 ± 0.19	1.82±0.43

In the observation group, ten days after the treatment, level 1 patients under the Wagner classification had the following results: 5 bloody vesicles in 14 limbs healed completely, 4 in 9 superficial ulcerations healed, and 5 had improved surrounding skin color and narrowed ulcer surface. For level 2 patients, the inflammation was reduced in 24 limbs. The purulent secretions were significantly reduced as well, and ulcers started to grow granulation tissues. For level 3 patients, the surrounding skin began to grow and purulent secretions and vomica were reduced in 17 limbs. For level 4 patients, wet and dry gangrene wounds were reduced and some necrotic tissues dropped off in 4 limbs. Thirty days after the treatment, the patients from levels 1 and 2 had their ulcers healed completely. For the patients from level 3, 14 ulcers healed, other ulcers no longer produced purulent secretions, the vomica became shallow, and the ulcer surface was reduced significantly. For the patients from level 4, the ulcer surface was reduced significantly, necrotic tissues dropped off, and new granulation tissues started to grow. Sixty days after treatment, all ulcers healed without any amputation.

### Complications and follow-up

The observation group had no complications, such as acute occlusion or distal vascular embolization. Four patients broke their anterior tibial artery or peroneal artery during the surgery, but the swelling and pain disappeared after application of cold compress, physical therapy, and Hirudoid ointment treatment. During the follow-up within one year to three and a half years, all patients did not get serious ischemia symptoms in their lower extremities. The patients who received infrapopliteal artery balloon expansion surgery had a patency rate of more than 80%. Moreover, the limb ulcer had already healed, and the clinical symptoms had disappeared. None of the patients received amputation surgery.

## Discussion

In the world, due to changes in the structure of people’s diet, the incidence of diabetes has increased gradually and so has incidences of lower extremity disability caused by diabetic peripheral arterial disease. The Framingham study showed that there was a 3.5- and 8.6-fold excess risk among men and women, respectively, of developing PAD in patients with DM [12].

Diabetic peripheral arterial disease is a characteristic of arteriosclerosis of the lower extremity arteries. It often involves several arteries of the double lower extremities [13,14]. Patients with diabetic peripathic arterial disease commonly show involvement of the arteries below the knee, especially at the tibial and peroneal arteries, and involvement of the profunda femoris [10]. It is multi-seasonally distributed and most prevalent in the tibial and peroneal arteries of the calf, including anterior tibial artery, posterior tibial artery and peroneal artery.

Life style modifications are the first mode of therapy, and most of the studies have shown physical exercise improves exercise tolerance at least a doubling in walking distance [15]. There is no conclusive evidence to suggest that optimal glycaemic control lowers the risk of PAD [16]. The management of dyslipidaemia in patients with diabetic peripheral arterial disease is warranted and the primary aim is LDL-cholesterol levels < 2.6 mmol⁄ l [17].

Due to the small diameter and the multi-branching of the arteries, the conventional treatment method of bypass surgery often has little effect on the stenosis or blockage. Furthermore, there was a high rate of postoperative restenosis and reblock, which is why the long-term outcome is not very satisfactory.

Therefore, with the constant updating of the treatment technology and equipment, the application of percutaneous transluminal angioplasty and luminal stenting surgery in the treatment of diabetes mellitus have caught the attention of surgeons at home and abroad. These techniques can radically improve the treatment of diabetic lower extremity arterial disease [18,19].

Since 2005, our department has carried out clinical research on endovascular treatment for diabetic peripheral arterial disease. Up until now, 81 patients have been treated. This study is currently in progress. The results showed that the clinical symptoms, vascular ultrasonic, and foot ulcer healing conditions of the observation group had significantly improved after the treatment. In contrast, the clinical symptoms, vascular ultrasonic, and foot ulcer healing conditions of the control group had no significant improvement after the treatment. There were no significant differences between the two groups before the treatment. Even if the observation group had a significant improvement compared with the control group after the treatment, all the above information showed only that endovascular treatment has a satisfactory short-term effect for diabetic lower extremity arterial disease. However, due to the limitation of the short follow-up time and the inadequate number of patients, the long-term efficacy of endovascular treatment remains unclear. It requires a long-term accumulation of information from a large sample.

Through this research, it can be concluded that endovascular treatment for diabetic peripheral arterial disease has many advantages: a) It has smaller chances of trauma and a faster recovery speed. It can also provide an immediate effect after the treatment; b) It has a high success rate and a low mortality rate. The current success rate of interventional therapy is up to 100%, while mortality rate is almost 0; c) It has a high rate of limb salvage. The diabetic foot ulcers caused by hypoxia-ischemia of patients who have undergone interventional treatment will gradually heal with the recanalization of blood vessels. As a result, the limb salvage rate was significantly higher than before; d) It is a kind of repeatable treatment. With a repeatable balloon dilatation, the restenosis of the lesion can be re-expanded with the same safety and effectiveness to improve the rate to save ischemic limbs; e) The intervention requires only local anesthesia, which has less side effects and is more suitable for elderly and frail patients [20].

One reported that patients with DM develop more symptomatic forms of PAD such as intermittent claudication, foot ulcers and critical limb ischaemia symptoms [21]. But in the follow-up, we found that patients who received infrapopliteal artery balloon expansion surgery have high restenosis rate, but the limb ulcer have healed, and the clinical symptoms have disappeared. Thus, the endovascular treatment for diabetic peripheral arterial disease could rapidly improve the blood supply and provide time for the foot ulcer and sectional toe wound to heal. With the gradual formation of restenosis, the collateral compensatory circulation was also gradually established, thereby greatly increasing the limb-salvage rate. These are the clinical significance and value of the endovascular treatment for diabetic peripheral arterial disease.

Because in the small diameter intravascular stent can lead to the early thrombosis and late lumen lost, thus affecting support long-term patency rate. It still have considerable controversy for whether the infrapopliteal arteries stented. At present scholars do not recommend the infrapopliteal arteries conventional implant the drug-uncoated stents, and bare metal stents is limited to postoperative failure cases of balloon expansion as a remedial method. With the new technology and new method development, some surgeon try to have drug-coated stents in the infrapopliteal arteries. Our groups of patients of infrapopliteal arteries had a good treatment effect after balloon expansion and no vascular rupture cases, so there is no implant any stents.

## Conclusions

Diabetic peripheral arterial disease is the main cause of lower limb amputation in patients with diabetes. We summarized the technique and evaluated the clinical effects of blood vessel intervention operation on diabetic peripheral artery disease. 81 cases of the observation group were treated by balloon PTA. We draw a conclusion that PTA is a feasible technique for diabetic peripheral artery disease. The endovascular treatment has a significant clinical effect in treating diabetic peripheral arterial disease. It can obviously improve the limb-salvage rate and the quality of life of the patients. With its smaller chances of trauma, higher safety, and fewer complications, it has great clinical significance in treating diabetic peripheral arterial disease. Although its short-term effects is satisfactory, the long-term effects is necessary for follow up.

## Competing interests

The authors declared no competing interest with respect to the authorship and/or publication of this article.

## Authors’ contributions

NF-Sun carried out the studies of Clinical trials and drafted the manuscript. YL-Tian participated collect and organize of data. L Xu performed the statistical analysis. AL-Tian and SY-Hu conceived of the study, and participated in its design and coordination and helped to draft the manuscript. All authors read and approved the final manuscript.

## Pre-publication history

The pre-publication history for this paper can be accessed here:

http://www.biomedcentral.com/1471-2482/13/32/prepub
